# The auditory cortex hosts network nodes influential for emotion processing: An fMRI study on music-evoked fear and joy

**DOI:** 10.1371/journal.pone.0190057

**Published:** 2018-01-31

**Authors:** Stefan Koelsch, Stavros Skouras, Gabriele Lohmann

**Affiliations:** 1 Department of Biological and Medical Psychology, University of Bergen, Bergen, Norway; 2 Department of Education and Psychology, Freie Universität Berlin, Berlin, Germany; 3 Department of Biomedical Magnetic Resonance, University Clinic Tübingen, Tübingen, Germany; 4 Magnetic Resonance Center, Max Planck Institute for Biological Cybernetics, Tübingen, Germany; University of Texas at Austin, UNITED STATES

## Abstract

Sound is a potent elicitor of emotions. Auditory core, belt and parabelt regions have anatomical connections to a large array of limbic and paralimbic structures which are involved in the generation of affective activity. However, little is known about the functional role of auditory cortical regions in emotion processing. Using functional magnetic resonance imaging and music stimuli that evoke joy or fear, our study reveals that anterior and posterior regions of auditory association cortex have emotion-characteristic functional connectivity with limbic/paralimbic (insula, cingulate cortex, and striatum), somatosensory, visual, motor-related, and attentional structures. We found that these regions have remarkably high emotion-characteristic eigenvector centrality, revealing that they have influential positions within emotion-processing brain networks with “small-world” properties. By contrast, primary auditory fields showed surprisingly strong emotion-characteristic functional connectivity with intra-auditory regions. Our findings demonstrate that the auditory cortex hosts regions that are influential within networks underlying the affective processing of auditory information. We anticipate our results to incite research specifying the role of the auditory cortex—and sensory systems in general—in emotion processing, beyond the traditional view that sensory cortices have merely perceptual functions.

## Introduction

Affective neuroscience has been interested primarily in limbic/paralimbic structures as neural correlates of emotion. Evidence regarding the role of sensory cortices in the incitement, regulation and modulation of emotions is relatively sparse. With regard to auditory processing, neuroanatomical studies showed projections from primary auditory cortex (PAC) to the lateral amygdala in rats [1], and it has been well established that these projections are involved in fear conditioning to auditory stimuli, and thus probably involved in the modulation of fear responses. However, the role of the auditory cortex in emotional responses to sounds is still only poorly understood.

Neurons of the PAC (also referred to as auditory core) mainly project to surrounding auditory belt fields which, in turn, mainly project to auditory parabelt regions [2, 3]. Areas located anterior to the auditory parabelt on the anterior superior temporal plane and the temporal pole (areas Pro, TS1 and TS2 according to Galaburda & Pandya [4]) host projections to the medial and orbital frontal cortex [5, 6], whereas anterior auditory belt and rostrally adjacent parabelt areas project to the anterior frontolateral cortex [5–8]. Posterior auditory fields (posterior belt and particularly posterior parabelt) host projections to the posterior frontolateral cortex [6, 9]. The posterior belt (and probably parabelt) areas receive somatosensory input via the adjacent retroinsular cortex and granular insula [10]. Moreover, the parabelt fields of the superior temporal sulcus (STS) project to numerous neocortical and limbic/paralimbic regions, in particular regions of the posterior parietal lobe, pre-occipital regions, cingulate, insular, parahippocampal, and medial paralimbic cortex [11–13]. Finally, auditory core, belt, as well as parabelt fields host projections to the striatum [14] as well as to the ento- and perirhinal cortex [15]. Thus, while it is clear that the auditory cortex hosts an abundance of anatomical connections with limbic and paralimbic brain structures, the functional significance of these projections is largely unknown.

One piece of evidence for the functional involvement of the auditory cortex in emotional processing is that the PAC is responsive to the sensory dissonance of acoustical stimuli [16], an acoustical feature that also elicits emotional reactions [17–19]. This suggests that the auditory cortex plays a role in the generation of pleasure/displeasure in response to sounds, perhaps in addition to the auditory brainstem, in which neuronal firing patterns also represent acoustical roughness [20] (but note that the preference of consonance over dissonance is strongly influenced by cultural experience [17]). Moreover, Peretz et al. [21] reported a patient with lesions to areas including the superior temporal gyrus (STG) bilaterally (in the right hemisphere, only the anterior STG was lesioned), the left middle temporal gyrus (MTG), and the bilateral insula. This patient did not have particular difficulties in recognizing linguistic prosody, but was impaired in interpreting the emotional tone conveyed by prosodic cues. Finally, the notion of a connection between the auditory cortex and emotional activity is supported by a plethora of functional neuroimaging studies that reported, e.g., activity differences in auditory core, belt, or parabelt regions during different emotion conditions using music [19, 22–29] (for a review see [30]) and affective vocalizations [31–36] (for a review see [37]). Nevertheless, there is consensus that auditory cortical regions involved in affective sound processing are still underspecified [37].

The present study addresses this issue by aiming to explore the functional connectivity between different auditory cortical regions and emotion-characteristic brain networks. Similar to previous studies [27, 29], we presented participants with music suited to evoke feelings of joy or fear. To determine candidate regions in the auditory cortex for interactions with emotion networks, we identified peak voxels of the contrasts joy vs. fear using both a traditional general linear model (GLM) approach and Eigenvector Centrality Mapping (ECM, see Methods for details). Then, these peak voxels were used as seed voxels in a Psychophysiological Interaction analysis (PPI) that compared the functional connectivity patterns of these seed voxels between the two different emotion conditions (joy and fear). Thus, this analysis aimed at identifying *emotion-characteristic functional connections* of different auditory regions, i.e., functional connections that are emotion-characteristic in that they are stronger during joy than fear, or vice versa. Based on previous functional neuroimaging studies (see above and [30, 37]), we expected to find emotion-characteristic regions in auditory core, belt, and parabelt regions. Based on the anatomical connections of these auditory regions (as reviewed above), we hypothesized that such emotion-sensitive regions would show emotion-characteristic functional connections with other auditory regions and with non-auditory regions, in particular amygdala, striatum, orbitofrontal, cingulate, insular, and entorhinal cortex, as well as frontolateral, parietal, and (pre-)occipital cortex.

## Materials and methods

### Participants

24 individuals (aged 19—31 years, *M* = 23.39, *SD* = 3.3, 12 females) took part in the experiment. All participants had normal hearing (as assessed with standard pure tone audiometry) and were right-handed (according to self-report). None of the participants was a professional musician or music student; 12 participants had no or only minimal formal musical training, and 12 participants were amateur musicians who had learned a musical instrument (five participants had learned a string instrument, three piano, three flute, and one participant had learned drums; mean duration of formal training was 4.7 years). Exclusion criteria were left-handedness, a score on Beck’s Depression Inventory (BDI) [38] of ≥ 13, past diagnosis of a neurological or psychiatric disorder, and abnormal brain anatomy, such as brain cysts identified during data acquisition. 18 of the 24 datasets were taken from a previous study [27]. All participants were students at the Free University of Berlin, were of German nationality and had a Western cultural background.

### Ethics statement

All subjects gave written informed consent. The study was conducted according to the Declaration of Helsinki and approved by the ethics committee of the School of Life Sciences and the Psychology Department of the University of Sussex.

### Stimuli and procedure

**Fig 1:**
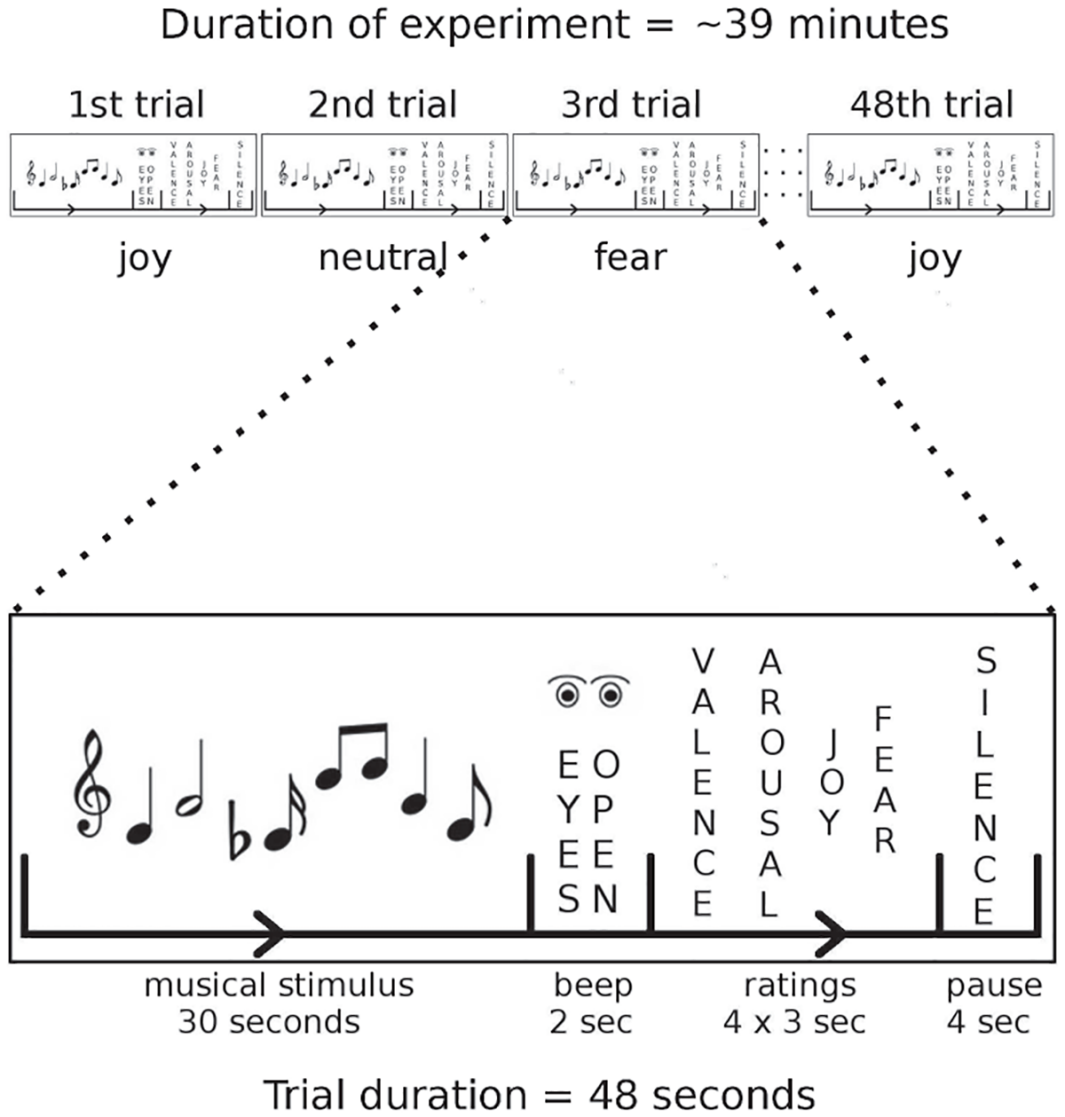
Experimental design. In each trial, a music stimulus was presented for 30 s. Music stimuli were pseudorandomly either a joy, a fear, or a neutral stimulus. Participants listened to the music with their eyes closed. Then, a beep tone signalled to open the eyes and to commence the rating procedure. Four ratings (felt valence, arousal, joy, and fear) were obtained in 12 s, followed by a 4 s pause (during which participants closed their eyes again). Trial duration was 48 s and the experiment comprised of 48 trials.

Stimuli and procedure were identical to a previous study [27] (see Fig 1 for an illustration of the experimental paradigm). Musical stimuli (each 30 s long) were selected to evoke (a) feelings of joy, (b) feelings of fear, or (c) neither joy nor fear (referred to as neutral stimuli). There were *n* = 8 stimuli per category. Joy stimuli consisted of CD-recordings from various epochs and styles (classical music, Irish jigs, jazz, reggae, South American and Balkan music). Fear stimuli were excerpts from soundtracks of suspense movies, TV series and computer games. The complete list of joy and fear stimuli is provided in S1 Table. Synthesis of neutral stimuli is described further below. All stimuli can be obtained online (http://stefan-koelsch.de/stimulus_repository/joy_fear_neutral_music.zip).

To further increase the fear-evoking quality of the musical stimuli, their acoustic roughness was increased electronically (for details see [27]). Importantly, stimuli were chosen in such a way that each joy excerpt was matched with a fear counterpart with regard to tempo (beats per minute), mean F0 pitch, pitch variation, and pitch centroid value (acoustic analysis of the stimuli was performed using ‘Essentia’, a library for extracting audio and music features from audio files, http://mtg.upf.edu/technologies/essentia). Parameters that differed significantly between joy and fear tunes were: dissonance, inharmonicity, key strength, diatonic strength, and pitch strength. Fear stimuli were more dissonant, featured more inharmonic sounds, their F0 pitch frequencies were less salient (due to more percussive sounds, and more hissing and whooshing noises), and pitches were less clearly attributable to tonal keys. Details of the statistical comparison of acoustic features between conditions is provided in S1 Text.

Neutral stimuli were sequences of isochronous tones, for which the pitch classes were randomly selected from a pentatonic scale. The neutral stimuli were designed following a procedure that we have previously described in detail [27]. Briefly, tone sequences were coded in MIDI (musical instrument digital interface) and generated using the MIDI toolbox for Matlab [39]. Importantly, for each joy-fear stimulus pair, a neutral control stimulus was generated that matched the joy and fear stimuli with regard to tempo, pitch range, and instrumentation (using the two respective main instruments or instrument groups of the respective joy-fear pair). To create stimuli that sounded like musical compositions played with real instruments (similar to the joy and fear stimuli), the tones from the MIDI sequences were set to trigger instrument samples from a high quality natural instrument library (X-Sample Chamber Ensemble, Winkler & Stahl GbR, Detmold, Germany) and from the Ableton Instrument library (Ableton Inc., New York, USA). Stimuli were then rendered as audio files using Ableton Live (version 8.0.4, Ableton Inc., New York, USA). The emotional neutrality of the stimuli was confirmed via a behavioural stimulus validation pilot study that involved emotional ratings. Only neutral stimuli that were consistently rated around the midpoint of Likert scales for valence, arousal, joy and fear were used in the main study.

Using Praat 5.0.29 [40], all music stimuli (joy, fear, and neutral) were edited so that they all (1) started at the beginning of a musical bar, (2) had the same length (30 s), (3) featured 1.5 s fade-in and fade-out ramps, and (4) featured the same acoustic power, as measured by the root mean square method for determining the average sound pressure.

Prior to the MRI session, participants were presented with short versions of each stimulus to obtain the familiarity of subjects with the stimuli. Participants rated their familiarity with each piece on a four-point scale, ranging from 1 (“To my knowledge I have never heard this piece before”) to 4 (“I know this piece, and I know who composed, or performed it”). None of the participants responded with “4” to any of the pieces, and a Kruskal-Wallis non-parametric one-way independent measures ANOVA performed using the software SPSS Statistics 19 (IBM Corporation, Armonk, U.S.A.) indicated that the average familiarity ratings did not differ (*p* = 0.87) between joy (*M* = .5, *SD* = 4.2), neutral (*M* = .6, *SD* = 4.1) and fear (*M* = .5, *SD* = 4.3) stimuli. Participants were then trained on the rating procedure (see below), using musical pieces that did not belong to the stimulus set used in the fMRI scanning session.

During the fMRI scanning session, stimuli were presented in pseudo-random order in a way that no more than two stimuli of each stimulus category (joy, fear, neutral) followed each other. Participants were asked to listen to the music with their eyes closed. Each music stimulus was followed by an interval of 2 s in which a beep tone of 350 Hz and 1 s duration signalled participants to open their eyes and to commence the rating procedure, during which they were asked to indicate how they felt at the end of each excerpt with regard to *valence* (pleasantness/unpleasantness), *arousal* (calm/excited), *joy* and *fear*. That is, participants provided ratings about how they felt, and not about which emotion each music stimulus was supposed to express. Ratings were obtained with 6-point Likert scales (ranging from “not at all” to “very much”), using an MRI-compatible response box with fiberoptic connectors (fORP 904 Subject Response Package, Cambridge Research Systems Ltd, Rochester, UK). The time interval for the rating period was 12 s. Each rating period was followed by a pause of 4 s, amounting to a total length of 48 s per trial (see Fig 1). The entire stimulus set (24 stimuli) was presented twice during the fMRI scanning session to increase the statistical power of the fMRI analysis, resulting in 48 trials and in an fMRI paradigm lasting 38,4 minutes (including rating and silence periods; see Fig 1). The entire scanning session included additional 10 s at the beginning of the experiment to allow for MRI field saturation and another 30 s after the end of the experiment, resulting in total fMRI scanning time of 39 minutes and 14 seconds.

Musical stimuli were presented using Presentation (version 13.0, Neurobehavioral systems, Albany, CA, USA) via MRI compatible headphones (under which participants wore earplugs). Instructions and rating screens were delivered through MRI compatible liquid crystal display goggles (Resonance Technology Inc., Northridge, CA, USA).

### MR scanning

Scanning was performed with a 3T Siemens TIM Trio (Siemens AG, Berlin, Germany) at the Dahlem Institute for Neuroimaging of Emotions (Berlin, Germany) between the years 2009 and 2010. Prior to the functional MR measurements, a high-resolution (1x1x1 mm) T1-weighted anatomical reference image was acquired from each participant using a rapid acquisition gradient echo (MP-RAGE) sequence. Continuous Echo Planar Imaging (EPI) was used with a TE of 30 ms and a TR of 2,000 ms. Slice-acquisition was interleaved within the TR interval. The matrix acquired was 64x64 voxels with a Field Of View (FOV) of 192 mm, resulting in an in-plane resolution of 3 mm. Slice thickness was 3 mm with an interslice gap of 0.6 mm (37 slices, whole brain coverage). The acquisition window was tilted at an angle of 30 degrees relative to the AC-PC line in order to minimize susceptibility artifacts in the orbitofrontal cortex [41–43]. We did not choose a sparse temporal scanning design in the present study because a primary interest was to apply ECM (see below), for which continuous fMRI data are better suited.

### Data analysis

FMRI data were processed and analysed using LIPSIA 2.1 [44]. Data were corrected for movement and slicetime acquisition and normalized into MNI-space-registered images with isotropic voxels of 3 cubic millimetres. A temporal highpass filter with a cutoff frequency of 1/90 Hz was applied to remove low frequency drifts in the fMRI time series, and a spatial smoothing was performed using a 3D Gaussian kernel and a filter size of 6 mm FWHM.

#### GLM analysis

A mixed effects block design GLM analysis [45] was employed and the realignment parameters were included in the design matrix as covariates [46]. One sample *t*−tests were calculated using the first-level contrasts between experimental conditions (i.e. joy vs. fear, joy vs. neutral, and fear vs. neutral). Results were corrected for multiple comparisons by the use of Monte-Carlo simulations implemented in LIPSIA, resulting in the identification of significant clusters (*p* < 0.05).

#### ECM analysis

**Fig 2:**
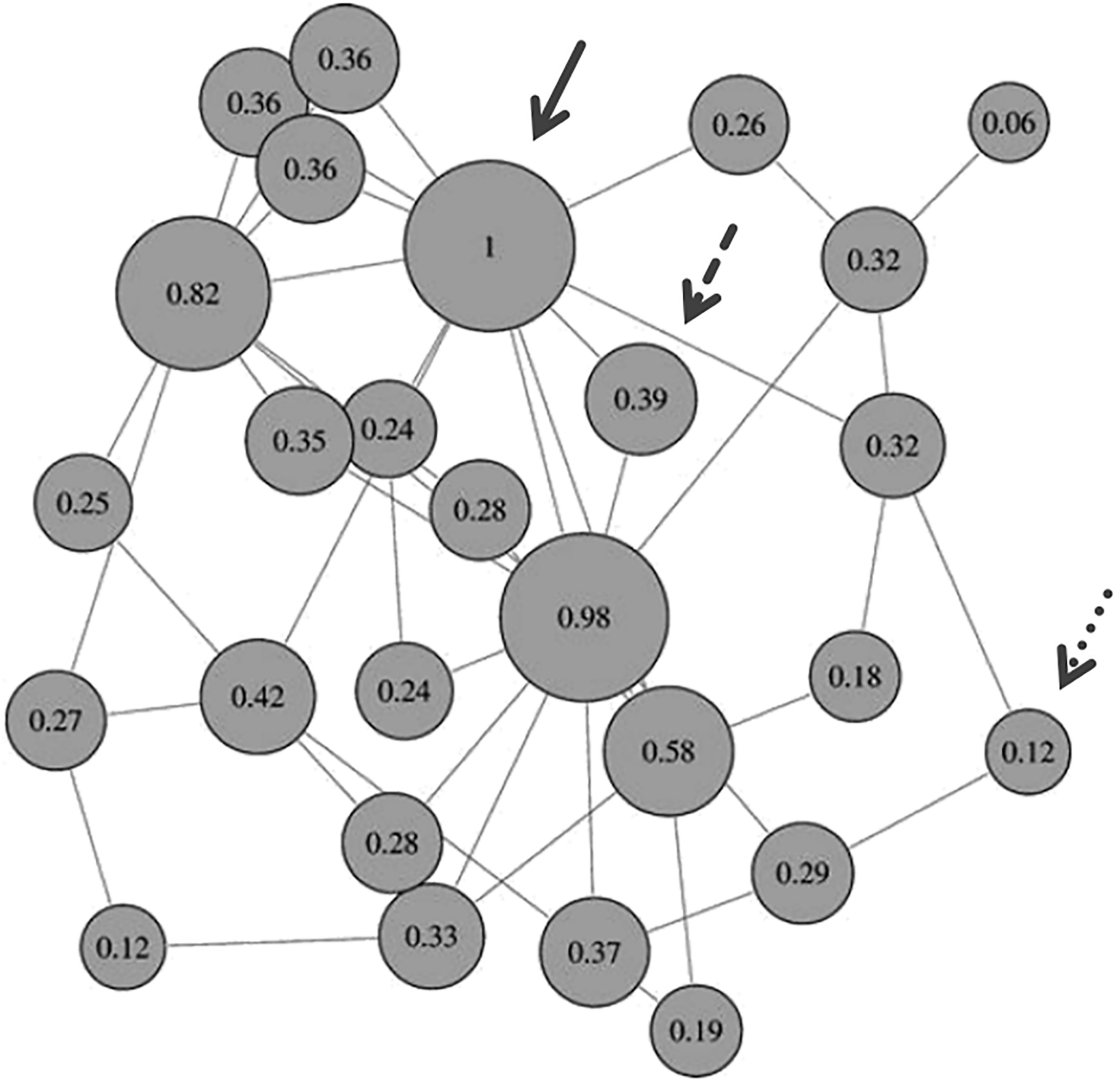
Illustration of eigenvector centrality. The figure illustrates a network with nodes (circles) and connections (lines). Some nodes are connected to several other nodes, whereas some nodes are only connected to two nodes, or even just one node (see the circle in the top-right corner). For each node, the eigenvector centrality value is indicated in the circle, and the circles are scaled in size according to their eigenvector centrality value. Note that eigenvector centrality does not only take the number of connections of a node into account, but also the importance of connected nodes. For example, the nodes indicated by the dashed and the dotted arrow both have two connections, but the node indicated by the dashed arrow has a higher centrality value because it is connected to the two nodes with the highest centrality values (the node with the highest centrality value is indicated by the solid arrow). ECM as applied in the current study treats each voxel as a node, and computes an eigenvector centrality value for each node (separately for each experimental condition, i.e. joy, fear, and neutral), thus identifying brain regions that are influential, or important within networks of functionally interconnected structures. Formulas for the computation of eigenvector centrality are provided in [47]. Reprinted and adapted with permission from [48].

Eigenvector Centrality Mapping (ECM) [47] computes a centrality value for each voxel in the brain such that a voxel receives a large value if it is strongly correlated with many other voxels that are themselves central within the network (for an illustration see Fig 2, for details see [47, 48]). Thus, ECM indicates influential, or important, computational hubs of neural networks with “small-world” properties in the human brain [49–51]. ECM can be applied to resting-state fMRI data, but it can also be computed for separate experimental conditions, such as different emotion conditions to explore different small-world networks underlying different emotions [29]. Hence, ECM can be used to identify emotion-characteristic computational hubs, beyond the computational hubs involved in resting state activity.

ECM was performed on the data obtained during the presentation of each stimulus (i.e., excluding ratings and rest intervals). To enable parametric statistical testing, eigenvector centrality values were transformed to have voxel-wise normal distributions across the sample, using a standard procedure [52] implemented as a LIPSIA built-in function [44]. Average eigenvector centrality maps were calculated for each condition and compared between all experimental conditions using paired t-tests. As for the GLM analysis, results were corrected for multiple comparisons by the use of Monte-Carlo simulations (*p* < 0.05).

#### PPI analysis

Psycho-Physiological Interaction (PPI) analyses were carried out to identify differences in the networks involved in the processing of joy compared with fear stimuli. According to our hypotheses and research aims, we restricted the PPI analyses to seed regions within the auditory cortex (as identified with the GLM and the ECM analyses). Seed voxels in the primary auditory cortex were determined based based on the GLM contrast *joy* > *fear*, and seed voxels for the planum polare and planum temporale were determined based on the ECM contrast *fear* > *joy* (see Results; for the approximate size of these and other brain structures see [53–55]). The coordinates of seed voxels were individually adjusted for the PPI analysis: For each participant, and for each structure identified in the group results, we identified the coordinate of the peak voxel of that participant within a sphere of 4 mm radius around the peak coordinate of the respective GLM, or ECM cluster. Then, functional connectivity analyses were conducted for all seed voxels (separately for joy and fear stimuli), and one-sample *t*−tests were computed to compare functional networks between experimental conditions. Tests were corrected for multiple comparisons by the use of Monte-Carlo simulations (*p* < 0.001) [47].

## Results

### Behavioural data

Behavioural data are provided in Table 1 and summarized in S1 Fig. *Valence (pleasantness) ratings* were higher for joy than neutral stimuli (*t*(23) = 12.70, *p* < 0.0001), higher for joy than fear stimuli (*t*(23) = 10.02, *p* < 0.0001), and did not differ significantly between fear and neutral stimuli (*p* > .1). *Arousal ratings* were higher for joy than neutral stimuli (*t*(23) = 6.63, *p* < 0.0001), higher for fear than neutral stimuli (*t*(23) = 5.79, *p* < 0.0001), and did not differ between joy and fear stimuli (*p* > 0.9). *Joy ratings* were higher for joy than neutral pieces (*t*(23) = 15.07, *p* < 0.0001), and higher for neutral than fear pieces (*t*(23) = 6.73, *p* < 0.0001). Correspondingly, *fear ratings* were higher for fear than neutral stimuli (*t*(23) = 8.10, *p* < 0.0001), and higher for neutral than joy stimuli (*t*(23) = 7.54, *p* < 0.0001).

**Table 1:** Emotion ratings provided by the participants.

	fear music	neutral music	joy music
valence	2.46 (0.80)	2.77 (0.63)	4.83 (0.62)
arousal	4.03 (0.78)	3.09 (0.71)	4.04 (0.60)
joy	1.72 (0.53)	2.50 (0.68)	4.87 (0.61)
fear	3.97 (0.95)	2.33 (0.83)	1.32 (0.36)

#### GLM contrast analysis

**Fig 3:**
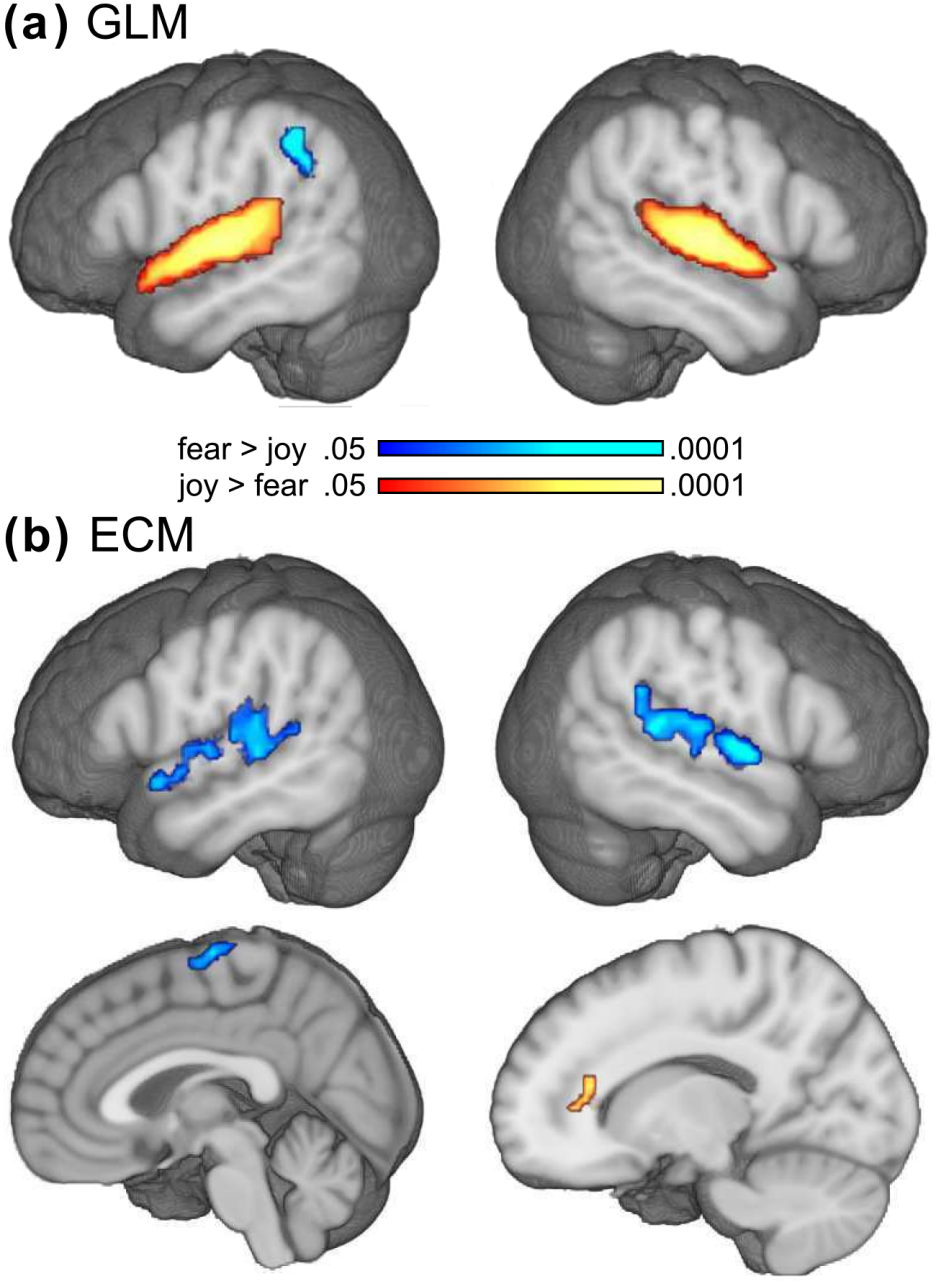
Results of the GLM and ECM analysis. Panel **a** shows the results of the general linear model (GLM) analysis, comparing BOLD signal intensity between joy and fear conditions. Higher BOLD signal intensity was measured in the auditory cortex bilaterally during joy compared with fear music (depicted in red-yellow colour), with peak voxels in the primary auditory cortex. The opposite contrast (fear > joy) indicated higher BOLD signal intensity in the left angular gyrus (depicted in blue). Panel **b** shows the result of the Eigenvector Centrality Mapping (ECM) analysis (scale is the same as in **a**). Higher centrality for fear than joy music (depicted in blue) was indicated in both anterior and posterior auditory regions, as well as in the paracentral lobule (bottom panel). No difference in centrality was observed in the primary auditory cortex. Joy stimuli (compared with fear stimuli) evoked higher centrality in the anterior cingulate cortex (depicted in yellow-red). Note that fear stimuli evoked higher centrality in the auditory cortex (as shown in **b**), whereas joy stimuli evoked stronger BOLD signal in the same areas (as shown in **a**). Results of both GLM and ECM analysis were corrected for multiple comparisons (*p* < .05).

Based on the general linear model (GLM), statistical parametric maps (SPMs) were computed separately for each condition, and compared between conditions using voxel-wise *t*−tests. Results of these tests are listed in Table 2 and shown in Fig 3a. The contrast *joy* > *fear* (red-yellow color in Fig 3a) showed significant activation of the supratemporal cortex bilaterally, extending laterally onto the convexity of the STG, and medially into the temporal operculum, with the maxima of activations being located in the primary auditory cortex on Heschl’s gyrus (TE1.0 according to the SPM anatomy toolbox [56]). The opposite contrast (*fear* > *joy*, blue color in Fig 3a) showed an activation within the (left) angular field of the inferior parietal lobule (IPL). Comparisons with the neutral condition showed that effects in the supratemporal cortex were due to an increase of BOLD signal during the joy condition, and a decrease of BOLD signal during the fear condition. Specifically, BOLD responses were stronger to joy than neutral stimuli in both (left) planum polare (p.p.) and (right) planum temporale (p.t.), and stronger to neutral than to fear stimuli in both left and right p.t. In the IPL, BOLD signal values did not differ significantly between joy vs. neutral, or fear vs. neutral. A previous study using the same experimental design [27] also reported bilateral activation of the amygdala for the contrast *joy* > *fear*. Although not significant in the corrected whole-brain analysis, there were local maxima in these structures at MNI coordinates -21 -9 -14 and 18 -9 -11 for the comparison *joy* > *fear*, and a region of interest analysis (using spheres with a 3 mm radius) showed that these signal differences were statistically significant (left: *p* = .003, right: *p* = .0003).

**Table 2:** GLM results of the comparisons between emotion conditions (joy, fear, neutral).

	MNI coord.	cluster size (mm^3^)	*z*−value: max (mean)
*Joy* > *fear*			
L Heschl’s gyrus	-51 -18 10	22788	7.33 (4.67)
R Heschl’s gyrus	51 -21 7	20952	7.33 (4.67)
*Fear* > *joy*			
L angular gyrus	-63 -48 31	1944	-4.08 (-3.40)
*Fear* > *neutral*			
L middle occipital gyrus	-27 -90 25	22599	5.26 (3.69)
R post. cuneus	18 -96 13	20520	5.47 (3.61)
L ant. cuneus	3 -69 16	405	3.65 (3.27)
L collateral sulcus / parahipp. G.	-36 -33 -11	1350	4.35 (3.46)
*Neutral* > *fear*			
L planum temporale	-66 -24 13	13473	6.63 (4.29)
R planum temporale	60 -24 10	15066	6.51 (4.28)
*Joy* > *neutral*			
L planum polare	-54 -3 1	13176	5.72 (4.04)
R planum temporale	57 -18 7	7749	5.99 (4.00)
L calcarine sulcus	-12 -90 1	11718	4.51 (3.47)

Abbreviations: ant.: anterior; G.: gyrus; L: left; parahipp.: parahippocampal; post.: posterior; R: right. All results were corrected for multiple comparisons (*p* < .05).

#### ECM analysis

Eigenvector Centrality Maps (ECMs) were computed separately for each condition, and compared between fear and joy conditions. Results of these contrasts are listed in Table 3 and shown in Fig 3b. The contrast *fear* > *joy* (blue colour in Fig 3b) showed clusters of voxels with significantly higher centrality values during fear (compared with joy) in the auditory cortex bilaterally. In the left hemisphere, the peak voxel was located in the planum polare, and another local maximum within this cluster was located in the planum temporale. In the right hemisphere the peak voxel was located in the planum temporale, and another local maximum within this cluster was located in the planum polare. In both hemispheres, these clusters extended laterally onto the convexity of the STG, and medially into the temporal operculum, but spared the primary auditory field (A1). Thus, in contrast to the GLM analysis, where joy music evoked higher BOLD signal intensity than fear music, the ECM analysis indicates higher centrality values for fear than joy music. Comparisons with the neutral condition showed that effects in the supratemporal cortex were due to an increase of centrality values during the fear condition, whereas neutral and joy conditions did not differ significantly from each other. Fig 3b also shows voxels with significantly higher centrality values during fear in the anterior paracentral lobule, i.e. the medial portion of Brodmann’s area 6 (caudal supplementary motor area), and higher centrality values during joy (compared with fear) in the (left) pregenual ACC (see red-yellow colours in Fig 3a).

**Table 3:** ECM results of the comparisons of centrality values between emotion conditions (joy, fear, neutral).

	MNI coord.	cluster size (mm^3^)	*z*−value: max (mean)
*Fear* > *joy*			
L planum polare	-51 -3 2	11151	4.41 (3.02)
L planum temporale^1^	-66 -30 22		4.02
R planum temporale	60 -24 14	14175	5.13 (3.28)
R planum polare^2^	54 -3 4		4.03
L paracentral lobule	-3 -15 73	675	3.84 (2.98)
*Joy* > *fear*			
L ACC	-12 30 13	540	3.88 (3.01)
*Fear* > *neutral*			
L planum temporale	-60 -15 10	16092	5.06 (3.34)
R planum temporale	60 -24 16	15930	5.55 (3.43)
R temporal pole	39 3 -14	432	3.43 (2.90)
L post. parahippocampal gyrus	-18 -33 -17	621	3.87 (2.98)
*Joy* > *neutral*			
cerebellar lobules IV/V	6 -54 -11	351	3.56 (2.97)

^1^ The cluster in the left aud. cortex had an additional local max. in the planum temporale.

^2^ The cluster in the right aud. cortex had an additional local max. in the planum polare.

Abbreviations: post.: posterior; L: left; R: right. All results were corrected for multiple comparisons (*p* < .05).

#### PPI analysis

**Fig 4:**
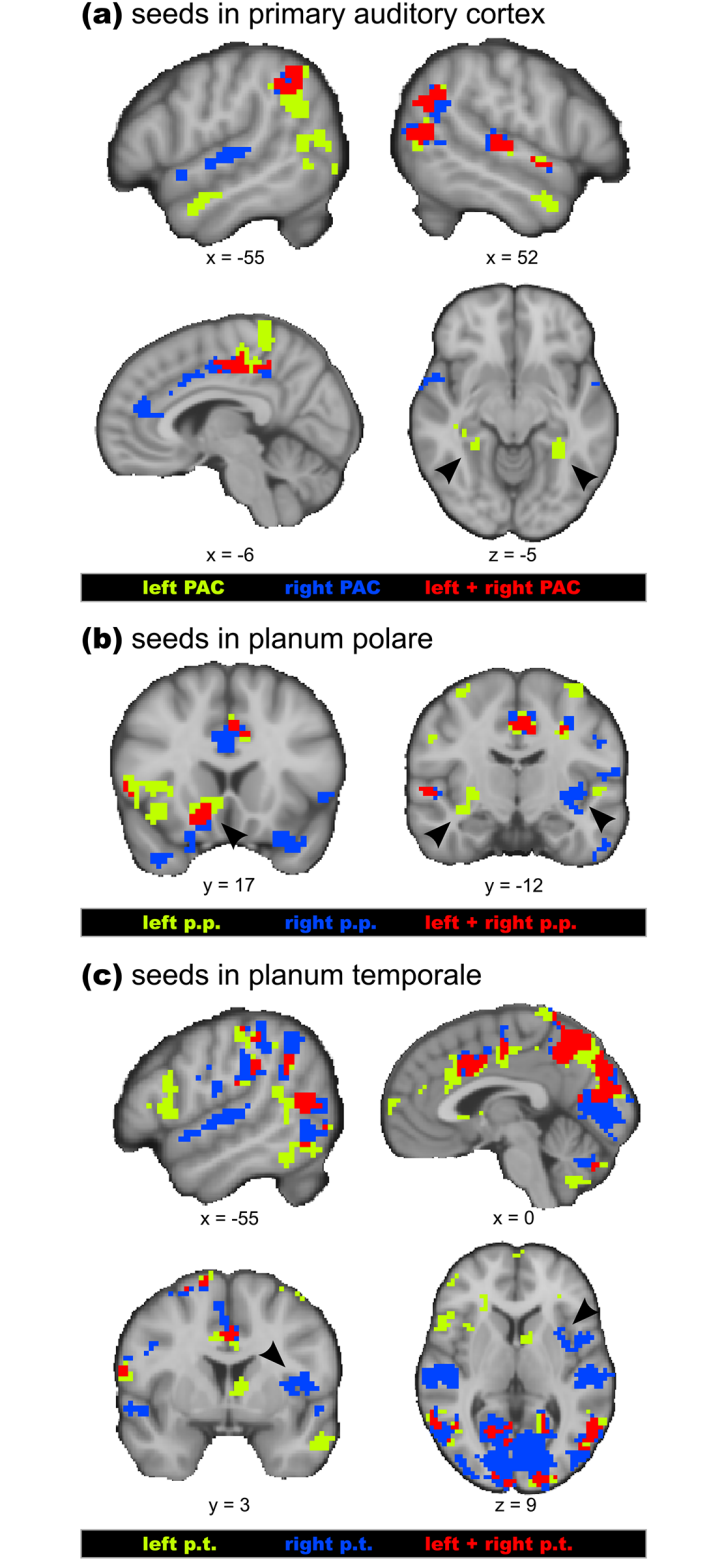
Summary of Psychophysiological Interaction (PPI) results. The figure shows conjunction analyses of emotion-characteristic functional connectivities for different auditory seed regions: Panel **a** shows the conjunction analysis of the PPI results for left and right primary auditory cortex (PAC) as seed regions, panel **b** shows the conjunction analysis of the PPI results for left and right planum polare (p.p.) as seed regions, and panel **c** shows the conjunction analysis of the PPI results for the left and right planum temporale (p.t.) as seed regions. Note that, for each seed region, a PPI analysis was computed. Then, regardless of whether the functional connectivity with a seed region was stronger during fear (compared with joy) or during joy (compared with fear), all significant results were included in the conjunction analysis. Red colour indicates voxels that showed emotion-characteristic functional connectivity with both left and right auditory regions, green colour indicates voxels that showed emotion-characteristic functional connectivity with left auditory regions only, and blue colour indicates voxels that showed emotion-characteristic functional connectivity with right auditory regions only (Tables 4, 5 and 6 provide information about whether the functional connectivity in each of these regions was stronger during fear compared with joy, or during joy compared with fear stimuli). Arrowheads indicate emotion-characteristic functional connectivity of left PAC with parahippocampal cortex bilaterally (panel **a**), of both left and right p.p. with the left ventral striatum (left of panel **b**), of the left p.p. with the left insula, and the right p.p. with the right insula (right of panel **b**), and of the right p.t. with right insular cortex (panel **c**).

The coordinates of peak voxels in the auditory cortex obtained in the GLM and the ECM analysis were used as seed regions for a PPI analysis (GLM joy > fear: left PAC -51 -18 10, right PAC: 51 -21 7; ECM fear > joy: left p.p. -51 -3 2, right p.p. 54 -3 4, left p.t. -66 -30 22, right p.t. 60 -24 14; note that, due to the individual adjustment of seed regions, as specified in the Methods section, taking seed coordinates for the p.p. or p.t. from the GLM contrasts involving the neutral condition leads to virtually identical results). Results of these analyses (corrected for multiple comparisons, *p* < 0.001), are provided in Tables 4, 5 & 6 and illustrated in Fig 4, which shows a conjunction analysis of the PPI results, separately for each pair of homotope auditory regions (primary auditory cortex, planum polare, planum temporale). Thus, Fig 4 also visualizes commonalities and differences in emotion-characteristic functional connectivity between left and right-hemispheric auditory regions (note that this conjunction analysis does not take into account whether functional connectivities were stronger during the fear or the joy condition, but see Tables 4, 5 & 6, in which positive *z*-values indicate stronger functional connectivity during joy than fear, and negative *z*-values stronger functional connectivity during fear than joy).

**Table 4:** PPI results with seeds in the primary auditory cortex.

Seed region	Functionally connected structures	MNI coord.	cluster size (mm^3^)	z-value: max (mean)
left primary auditory cortex				
	R p.t.	60 -24 7	2943	4.88 (3.62)
	R ant. STS	54 3 -23	972	-4.32 (-3.58)
	L ant. STS	-51 3 -20	1539	-4.16 (-3.44)
	L IPL	-60 -48 37	4401	-4.67 (-3.54)
	R TPO	51 -63 19	3834	-4.31 (-3.47)
	L TPO	-51 -54 10	2079	-4.19 (-3.38)
	PCC	-9 -21 49	5643	-4.72 (-3.53)
	L post. PHC	-27 -42 1	567	-3.82 (-3.35)
	R post. PHC	30 -45 -5	972	-4.66 (-3.54)
	PCL	-9 -39 76	3402	-4.93 (-3.52)
right primary auditory cortex				
	R p.t.	57 -24 10	3888	5.58 (3.95)
	L HG / p.t.	-60 -15 7	4077	4.75 (3.73)
	L IPL	-54 -54 52	2214	-4.23 (-3.48)
	R IPL	48 -60 37	5103	-4.97 (-3.58)
	R SFS	33 24 43	2241	-4.34 (-3.51)
	L SFS	-30 33 49	864	-4.89 (-3.72)
	ACC	-15 42 22	4239	-4.58 (-3.48)
	PCC	-9 -21 49	6156	-4.89 (-3.51)

Positive z-values indicate stronger functional connectivity between a seed region and a functionally connected structure during the joy (compared with the fear) condition, negative z-values indicate stronger functional connectivity between a seed region and a functionally connected structure during the fear (compared with the joy) condition. Abbreviations: ACC: anterior cingulate cortex; ant.: anterior; HG: Heschl’s gyrus; IPL: inferior parietal lobule; L: left; PCC: posterior cingulate cortex; PCL: paracentral lobule; PHC: parahippocampal cortex; post.: posterior; p.t.: planum temporale; R: right; SFS: superior frontal sulcus; STS: superior temporal sulcus; TPO: temporo-parieto-occipital area. All results were corrected for multiple comparisons (*p* < .05).

**Table 5:** PPI results with seeds in the planum polare.

Seed region	Functionally connected structures	MNI coord.	cluster size (mm^3^)	z-value: max (mean)
left planum polare				
	L HG / p.t.	-45 -27 13	2511	6.18 (3.62)
	R HG / p.t.	51 -27 13	3537	5.17 (3.74)
	MCC	-3 12 43	3240	-4.88 (-3.56)
	L ant. insula	-39 15 -5	3348	-4.95 (-3.48)
	L inferior circular s.	-36 -15 -8	1485	-5.23 (-3.59)
	L OFC	-22 37 -22	2608	-3.6 (-3.37)
	L post. SFS	-27 3 64	3186	-4.82 (-3.56)
	L SFM	-39 48 28	1890	-3.93 (-3.39)
	R SFM	36 48 25	1539	-4.14 (-3.36)
	R PCS / PMC	33 -9 73	2808	-4.87 (-3.67)
	L fusiform g.	-39 -39 -23	66852	-5.08 (-3.48)
	R TPO	63 -54 31	4455	-5.04 (-3.53)
	precuneus	-6 -66 67	19413	-5.11 (-3.50)
	R ant. occipital s.	48 -75 7	729	-3.61 (-3.30)
	L caudate n. (head)	-18 21 -5	2408	-5.46 (-3.57)
	R cerebellum	21 -42 -47	918	-4.43 (-3.51)
right planum polare				
	L HG /p.t.	-45 -24 10	4725	4.69 (3.36)
	R HG /p.t.	51 -27 10	2997	4.43 (3.28)
	L temporal pole	-27 9 -26	17712	-4.72 (-3.11)
	MCC	-9 -3 52	361206	-5.18 (-3.11)

Positive z-values indicate stronger functional connectivity between a seed region and a functionally connected structure during the joy (compared with the fear) condition, negative z-values indicate stronger functional connectivity between a seed region and a functionally connected structure during the fear (compared with the joy) condition. Abbreviations: ant.: anterior; g.: gyrus; HG: Heschl’s gyrus; L: left; MCC: middle cingulate cortex; n.: nucleus; OFC: orbitofrontal cortex; PCS: precentral sulcus; PMC: premotor cortex; post.: posterior; p.t.: planum temporale; R: right; s.: sulcus; SFM: sulcus frontomarginalis; SFS: superior frontal sulcus; SMA: supplementary motor area; TPO: temporo-parieto-occipital area. All results were corrected for multiple comparisons (*p* < .05).

**Table 6:** PPI results with seeds in the planum temporale.

Seed region	Functionally connected structures	MNI coord.	cluster size (mm^3^)	z-value: max (mean)
left planum temporale				
	MCC	3 21 37	4887	-4.53 (-3.43)
	PCC	9 -21 52	3537	-5.04 (-3.54)
	R OFC	45 24 -8	5751	-5.27 (-3.61)
	L MFG	-36 30 49	783	-4.08 (-3.43)
	R SFM	24 36 16	14796	-4.95 (-3.49)
	L IFG	-51 48 -2	2025	-5.02 (-3.76)
	L PCS / PMC	-15 0 76	594	-4.21 (-3.48)
	L PCS / PMC	36 -9 70	1836	-4.56 (-3.49)
	R TPO	54 -51 16	12393	-4.66 (-3.49)
	L occipital g.	-48 -66 -2	21249	-5.17 (-3.50)
	fusiform g.	36 -51 -20	3753	-4.27 (-3.43)
	cuneus	-15 -87 46	54162	-5.06 (-3.58)
	L cerebellum	-33 -81 -26	3510	-4.43 (-3.46)
	R cerebellum	30 -48 -47	4077	-4.72 (-3.46)
	cerebellar vermis	-3 -63 -41	3456	-5.72 (-3.62)
	L cerebellum	-21 -42 -50	1404	-3.93 (-3.37)
	R caudate n. (head)	-12 21 -8	10611	-4.67 (-3.60)
right planum temporale				
	L HG / p.t.	-48 -24 10	6237	5.69 (3.83)
	R HG / p.t.	51 -24 13	4725	4.81 (3.73)
	MCC	5 20 33	31295	-4.78 (-3.42)
	R post. insula	36 -6 1	3348	-4.25 (-3.41)
	L post. PHC	-14 -43 -6	18375	-4.67 (-3.59)
	L calcarine sulcus / V1^1^	-9 -72 13	64315	-5.59 (-3.59)
	precuneus	8 -65 67	28652	-4.57 (-3.48)
	cerebellar vermis	-3 -66 -29	1161	-4.38 (-3.41)

^1^ The cluster with the peak value in V1 had additional local maxima in V2—V5.

Positive z-values indicate stronger functional connectivity between a seed region and a functionally connected structure during the joy (compared with the fear) condition, negative z-values indicate stronger functional connectivity between a seed region and a functionally connected structure during the fear (compared with the joy) condition. Abbreviations: ant.: anterior; g.: gyrus; HG: Heschl’s gyrus; IFG: inferior frontal gyrus; L: left; MCC: middle cingulate cortex; MFG: middle frontal gyrus; n.: nucleus; OFC: orbitofrontal cortex; PCC: posterior cingulate cortex; PCS: precentral sulcus; PHC: parahippocampal cortex; PMC: premotor cortex; post.: posterior; p.t.: planum temporale; R: right; s.: sulcus; SFM: sulcus frontomarginalis; TPO: temporo-parieto-occipital area. All results were corrected for multiple comparisons (*p* < .05).

Both left and right *primary auditory cortex* (PAC) showed stronger emotion-characteristic functional connectivity with right auditory belt and parabelt regions (stronger during joy than fear, see Table 4 and red colour in Fig 4a). Moreover, both left and right PAC showed stronger functional connectivity with the inferior parietal lobule bilaterally, and with posterior cingulate cortex (all stronger during fear than joy, see Table 4 and red colour in Fig 4a). Right (but not left) PAC showed stronger functional connectivity with left auditory belt regions (during joy compared with fear), and with the anterior cingulate cortex (during fear compared with joy). Left (but not right) PAC showed stronger functional connectivity with the anterior STS bilaterally, and with the posterior parahippocampal cortex bilaterally (all stronger during fear compared with joy, see also arrowheads in Fig 4a).

Both left and right *planum polare* (p.p.) showed emotion-characteristic functional connectivity (stronger during fear than joy) with anterior cingulate cortex and the left ventral striatum / nucleus accumbens (Table 5 and red colour in Fig 4b). Moreover, both left and right p.p. showed ipsilateral connectivity with the posterior insula (stronger during fear than joy, see also arrowheads in the right panel of Fig 4b), and the left p.p. also showed emotion-characteristic functional connectivity with the left anterior insula (stronger during fear than joy).

Both left and right *planum temporale* (p.t.) showed emotion-characteristic functional connectivity (stronger during fear than joy) with the cuneus (visual cortex, V1—V5, see also red colour in Fig 4c) and precuneus, cingulate cortex, and ipsilateral pars opercularis of the IFG (see also Table 6). Moreover, the right p.t. showed emotion-characteristic functional connectivity (stronger during joy than fear) with the right anterior insula (see arrowheads in Fig 4c) and with supratemporal cortex bilaterally.

## Discussion

### Psychophysiological Interactions

The PPI results indicate that the auditory cortex hosts both provincial hubs with emotion-characteristic functional connections between auditory regions, and connector hubs with emotion-characteristic functional connections with limbic/paralimbic, visual, somatosensory, and motor systems. emotion-characteristic functional connectivity was observed (a) within the auditory cortex (i.e. between both ipsi- and contralateral auditory areas), (b) between auditory cortex and limbic/paralimbic structures (cingulate, insular, parahippocampal, and orbitofrontal cortex, as well as ventral striatum), and (c) between auditory cortex and extra-auditory neocortical areas (mainly visual, somatosensory, and motor areas).

The primary auditory cortex (PAC) mainly showed intrinsic (auditory-auditory) emotion-characteristic functional connections, either with contralateral PAC or with extra-primary auditory fields. This is well in accordance with previous literature on intrinsic auditory connections [3, 54]. Functional connections of the PAC were also observed with multisensory structures (such as the temporal parietal occipital area, TPO) and limbic/paralimbic structures (such as cingulate cortex and parahippocampal cortex). However, whether these connections truly originate from the PAC or from (directly adjacent) auditory belt fields is uncertain, given the spatial resolution of our study. Functional connectivity of PAC with extra-auditory regions would be consistent with previous anatomical evidence showing neural projections of the auditory cortex with non-auditory sensory and multisensory structures [10, 57–60].

With regard to the auditory association cortex, many of the emotion-characteristic functional connections observed in our study parallel anatomical connections previously described in monkeys (as reported below). Importantly, our results provide information about the emotion-characteristic nature of such connections (in humans). For example, in rhesus monkeys, the anterior and middle parts of the superior temporal plane project to the ventral striatum (to both the ventral head of the caudate and the ventral putamen [14]). In addition, several neurons in the ventral striatum respond to auditory stimuli when such stimuli are cues for specific movements, such as approach to appetitive, or withdrawal to aversive stimuli (for an overview see [14]). In the present study, the functional connectivity between the (left) planum polare (p.p.) and the (left) ventral striatum was stronger during fear than during joy music, perhaps because auditory signals of threat have strong behavioural relevance for immediate survival. The functional connectivity of the (left) p.p. with cortical regions along the (left) orbital sulcus of the orbitofrontal cortex (OFC) during the fear (compared with the joy condition) parallels findings of projections (in rhesus monkeys) from the anterior superior temporal plane to the OFC [5]. The OFC region observed in our study has been associated with the evaluation of negative reinforcers (“punishers”) that can lead to a change in behaviour [61]. In our study, emotion-characteristic functional connectivity of the p.p. with the OFC (during fear-evoking, unpleasant music) was thus probably due to the evaluation of the negatively valenced music, and perhaps also due to motor preparation, or evocation of motor alertness in the face of the fear-evoking stimuli. The notion of auditory-motor interactions is also supported by the functional connectivity between auditory regions and sensorimotor-related cortical regions (IPL, SMA and PMC). With regard to functional connections to the insula, our results parallel connections between the planum temporale (p.t.) and granular insula in macaque monkeys [10], taken as a likely source of somatosensory input into the auditory cortex [10]. Note that in (macaque) monkeys only very few connections exist between the anterior superior temporal plane and the insula [62]. Our results indicate clear functional connectivity between p.p. and the (agranular) anterior insula in humans, likely reflecting further sensory-limbic interactions. Such sensory-limbic interactions are also apparent in the functional connectivity of both anterior and posterior superior temporal plane (p.p. and p.t.) with the ACC. Finally, the PPI results also showed marked functional connectivity between auditory areas (both p.p. and p.t.) with the visual cortex (V1—V5). Anatomical results indicate that core, belt and parabelt regions project to V1 and V2 of the visual cortex, and that neurons in V2 project back into these auditory regions (reviewed in [10]). The observed functional connectivity between these areas in the present study highlights the role of auditory-visual interactions, in particular during emotional states of fear. The functional significance of such interactions is perhaps increased visual alertness.

Given that the seed regions for the PPI analysis in the p.p. and the p.t. had central, influential positions within affective brain networks (as indicated by the ECM contrasts), the PPI results indicate that the auditory association cortex host central hubs within emotion networks that are far more extensive than previously believed, involving functional connectivity with a diverse range of limbic/paralimbic as well as neocortical (extra-auditory sensory and motor) structures. This finding shows that the auditory cortex plays a central role in affective processes, in addition to its classical role in auditory perception (for a review of brain structures generating emotions see e.g. [63]). Moreover, this finding argues for the notion that multisensory interactions in the cerebral cortex are not limited to established polysensory regions, but also encompass sensory areas including the auditory cortex [10]. In particular, the emotion-characteristic functional connections between auditory cortex and insular cortex, as well as between auditory cortex and cingulate cortex, include interactions between auditory and limbic-sensory (interoceptive) cortex.

### Differences between ECM and GLM results

Another interesting finding of the present study is a striking difference between results obtained with the traditional general linear model (GLM) approach and ECM results. The GLM analysis showed stronger BOLD signal intensity during joy than fear in the auditory cortex (yellow-red colours in Fig 3a) By contrast, the ECM results indicated higher centrality values (i.e., more influential, or important positions in a network of functionally interconnected structures) in the same areas during fear compared with joy (blue colours in Fig 3b). This reveals that BOLD signal contrasts and ECM contrasts can indicate substantially different patterns of brain activity (in part within the same volume of interest), owing to the fact that the results of these two analysis methods reflect, in part, different neural functions. Whereas the magnitude of BOLD responses within a voxel is assumed to correlate with the amount of neural activity, the magnitude of the centrality value of a voxel correlates with the importance, or influence of this voxel within a network of interconnected brain structures. Because regional neural activity is not necessarily correlated with the influence of this region within a network, GLM and ECM results might reveal different patterns of brain activity, and thus yield complementary information about brain activity.

Traditionally, the inference from GLM contrasts is that areas showing stronger BOLD response in a certain condition are “activated”, “more important”, or “more strongly involved” in the processing of this condition. The present results suggest that such inferences about brain activations based on GLMs should be revisited, because they might have captured only one aspect of relevant brain activity: While a specific area might show stronger BOLD response during one experimental condition, it might show stronger network centrality, and stronger functional connectivity, during another. For example, while the regional neural activity in anterior and posterior auditory regions (p.p. and p.t.) was stronger during the joy than the fear condition (as indicated by the contrast of BOLD signals, see Fig 3b), the network centrality of these regions (i.e., the influence of these regions within a network of interconnected brain structures) was stronger during the fear than the joy condition (as indicated by the ECM contrasts, see Fig 3a). Perhaps fear involves faster, and stronger functional coordination of the auditory cortex with rapid fight and flight mechanisms (where the focus is rather on coordinated responses than detailed acoustical analysis), at least during the early stages of auditory processing. By contrast, the stronger BOLD signals during music-evoked joy might reflect stronger regional activity within the auditory cortex, probably due to a voluntary shift of attention towards the joy stimuli (participants had a preference for the joy stimuli, as indicated by the valence ratings, as in [27]). This notion is supported by the PPI results, showing increased functional connectivity of auditory areas during joy only with other auditory areas.

Note that, with regard to both GLM and ECM contrasts, differences between fear and joy were not due to the tempo of stimuli (in terms of beats per minute), neither due to mean F0 pitch, pitch variation, nor pitch centroid value (all of these factors were matched between joy, fear, and neutral stimuli). Dissonance and inharmonicity were stronger for the fear than joy excerpts, and stronger for joy than neutral excerpts (see S1 Text). However, neither the GLM nor the ECM results indicated a result corresponing to this pattern (*fear* > *joy* > *neutral* or vice versa) in any brain structure. Likewise, with regard to chord strength and mean F0 salience, no systematic associations were found with the GLM, or ECM results, and therefore it is unlikely that these acoustical factors contributed to the results observed in the present study.

### Duration of stimuli

It is noteworthy that the duration of the stimuli used in the present study was only 30 s, and that no significant differences in centrality were observed in the auditory cortex in a previous study using 4-minute blocks of joy and fear music using very similar stimuli [29]. Thus, the pattern of neural activity and functional connectivity observed in the present study holds for the initial stages of stimulus processing, and appears to change soon thereafter. Such temporal dynamics of neural activity in response to auditory stimuli with emotional valence is consistent with previous findings showing that the processing of pleasant music as opposed to unpleasant music has a different timecourse of neural activation [19, 27, 64], and that neural activation associated with the anticipation of intense music-evoked pleasure changes during the actual experience of such pleasure [65].

### Limitations and future directions

Our study has several limitations, some of which give rise to interesting new research directions: (1) We only used music evoking joy or fear. Thus, our results are likely not exhaustive, and it is possible that music evoking other emotions (e.g. sadness), or other auditory stimuli with affective valence (such as affective vocalizations, affective prosody, or non-human environmental sounds), are associated with additional functional connections between auditory regions and limbic/paralimbic brain structures. For example, it is likely that other sound stimuli (especially human affective vocalizations) will reveal functional connectivity of auditory parabelt regions (e.g., in the superior temporal gyrus, STS) with limbic/paralimbic structures. Note, however, that recent research provides strong arguments for the view that affective information of sounds is processed in common neural networks, rather than in “distinct neural systems for specific affective sound types” [37]. Following this unifying neural network perspective, it is likely that the results reported in the present study are not specific for music. (2) We did not systematically assess visual imagery, but recommend to do so in future studies. We observed that, in response to an open question of our post-imaging questionnaire that asked for participants’ experiences during the experiment, 12 participants reported visual imagery during both joy and fear music, and one participant reported visual imagery during fear but not joy music. These participants typically reported that they imagined “situations fitting to the music”, “film scenes fitting to the music”, “eerie things during the eerie music”, and “happy things during the happy music”, “a haunted house” or “monsters” during fear music, and “people partying” or “people dancing” during joyful music. Assessing visual imagery of participants in experiments on music and emotion can also further illuminate visual imagery as an important mechanism underlying the evocation of emotions with music, as e.g. suggested in the BRECVEMA model by Juslin [66] (see also the Imagination principle in [67]). (3) Our study sample did not include musicians, thus not allowing for the investigation of any effects that professional musical training might have on the role of the auditory cortex within emotional brain networks. (4) Our study did not address possible sex differences in emotion processing. Future studies might also use music to investigate this issue, e.g. with regard to emotional memories or emotion regulation. (5) A further limitation is the possibility that the emotion contrasts have been influenced by the valence of stimuli, or by psychoacoustical factors (e.g. sensory dissonance). However, both low valence and dissonance are important attributes of fear-evoking auditory stimuli, and such a possibility would not have a drastic impact on the comparison between PPI results from different seed regions (which comprise the main results of this study). (6) A valuable future research topic would be to functionally map the subfields of the auditory cortex using 7T-fMRI (e.g. using the mapping method employed by Petkov et al. [55]) and then specify within-subjects emotion-characteristic connections of these subfields. Based on our results, a-priori hypotheses can be formulated for target regions of interest, such as insula, cingulate cortex, striatum, and temporal pole.

## Conclusion

Fear stimuli (compared with joy stimuli) evoked higher network centrality in both anterior (planum polare) and posterior (planum temporale) auditory association cortex. This indicates that the auditory cortex hosts emotion-characteristic computational hubs within neural networks with “small-world” properties, and that the auditory cortex plays a central role in the affective processing of auditory information. With regard to their emotion-characteristic functional connectivity, primary auditory areas showed strong intra-auditory functional connectivity. Anterior and posterior auditory association cortex showed a range of emotion-characteristic functional connections with limbic/paralimbic structures (insula, striatum, cingulate cortex and orbitofrontal cortex) as well as with neocortical areas (visual cortex, precuneus, and inferior fronto-lateral cortex). Taken together, the present findings show that the auditory cortex hosts regions that are central relays in emotion networks that are more extensive than previously believed, featuring widespread emotion-characteristic connections between auditory areas and limbic/paralimbic structures, as well as between auditory and non-auditory neocortical areas. Thus, our results indicate that, beyond mere acoustical analysis, the auditory cortex plays a central role in the emotional processing of sounds.

## Supporting information

S1 Fig Behavioral ratings. Behavioral ratings provided by participants on the four emotion scales used in the present study: (a) valence, (b) arousal, (c) joy, and (d) fear. Ratings are depicted separately for each stimulus category (fear, neutral, joy).
(PDF)

S1 Text Statistics of acoustical features that differed between conditions. (PDF)

S2 Text PPI results for non-auditory seed regions. (PDF)

S1 Table List of stimuli. (PDF)

S2 Table PPI results for non-auditory seed regions. (PDF)
