# Supplementary material for: The auditory cortex hosts network nodes influential for emotion processing: An fMRI study on music-evoked fear and joy
Source: PLoS One. 2018 Jan 31;13(1):e0190057. doi: 10.1371/journal.pone.0190057 (PMC5791961; doi:10.1371/journal.pone.0190057)
Supplement: S1 Fig — Behavioral ratings provided by participants on the four emotion scales used in the present study: (a) valence, (b) arousal, (c) joy, and (d) fear. Ratings are depicted separately for each stimulus category (fear, neutral, joy). (PDF) [file pone.0190057.s001.pdf]

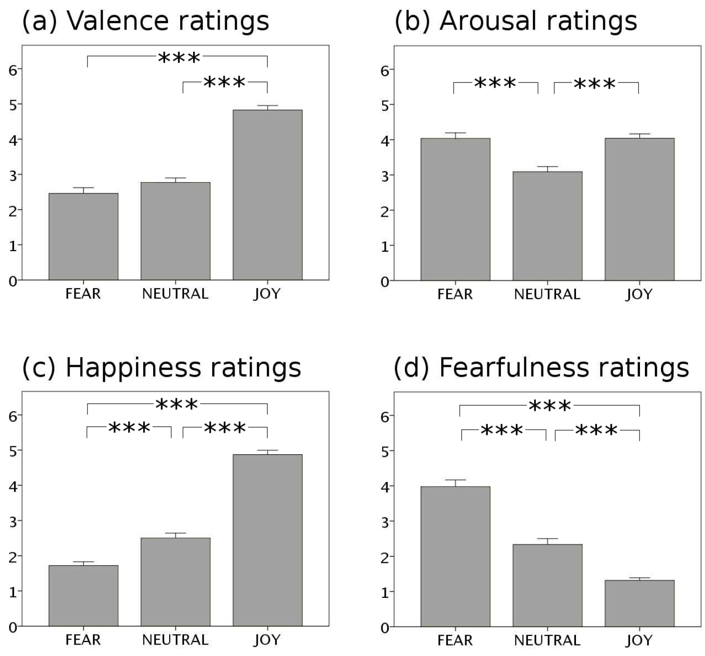

S1 Fig. Behavioral ratings. Behavioral ratings provided by participants on the four emotion scales used in the present study: (a) valence, (b) arousal, (c) joy, and (d) fear. Ratings are depicted separately for each stimulus category (fear, neutral, joy).
