# Supplementary material for: The auditory cortex hosts network nodes influential for emotion processing: An fMRI study on music-evoked fear and joy
Source: PLoS One. 2018 Jan 31;13(1):e0190057. doi: 10.1371/journal.pone.0190057 (PMC5791961; doi:10.1371/journal.pone.0190057)
Supplement: S1 Text — (PDF) [file pone.0190057.s002.pdf]

S1 Text. Statistics of acoustical features that differed between conditions. Using the Essentia software (http://mtg.upf.edu/technologies/essentia, see also main text) 177 acoustical descriptors were extracted frame-by-frame (frame length = 21.5 ms, 50% overlap), averaged along the entire duration of the file, and then compared between conditions (joy, neutral, fear) using one-way ANOVAs. Bonferroni-corrected significance-level was 0.05 / 177 = 0.00028 (lowering this threshold for one-sided tests, i.e. 0.00056, did not change any of the results). Significant effects of condition were indicated for the following acoustic factors (with $F-$values in parentheses, degrees of freedom: 2, 21): (a) Mean (72.3) and variance (13.8) of *F0 salience* (this measure is highest for single tones, intermediate for chords, and lowest for noises; note that mean F0 and variance of F0 did not differ between joy, fear, and neutral stimuli). The mean F0 salience was highest for neutral, intermediate for joy, and lowest for fear stimuli ($p < .0001$ in all pairwise comparisons). This reflects that both joy and fear (but not neutral) stimuli contained numerous harmonies, and that fear (but not joy) stimuli contained numerous percussive sounds, as well as hissing and whooshing noises. (b) Mean (41.3) and variance (28.0) of *sensory dissonance.* Sensory dissonance was lowest for neutral, intermediate for joy, and highest for fear stimuli. Mean sensory dissonance differed significantly between joy and neutral ($p < .0001$), between fear and neutral ($p < .0001$), and between joy and fear stimuli ($p < .05$). (c) Mean *chord strength* (25.2) and *key strength* (14.7); these factors measure how strongly a sound resembles the sound of a chord, and how clearly the sounds of a stimulus can be attributed to a key. Chord strength was higher for joy compared to fear stimuli ($p < .0001$), as well as for joy compared to neutral stimuli ($p < .0006$), whereas fear and neutral stimuli did not differ significantly from each other. Key strength was higher for joy compared to fear stimuli ($p < .0001$), and for neutral compared to fear stimuli ($p = .01$); joy and neutral stimuli did not differ significantly from each other ($p > .15$). (d) Mean (30.0) and variance (16.4) of *spectral flux* (a measure of spectral variation within sounds), mean (30.0) *spectral crest* (a measure of the inhomogeneity, or noisiness, of the spectrum) and mean (10.6) *spectral complexity* (which correlates with the amount of different timbres that are present in a piece). Mean spectral flux, spectral crest, and spectral complexity were lowest for neutral stimuli (with significant differences between neutral and joy, as well as between neutral and fear stimuli, $p < .05$ in each test), and did not differ significantly between joy and fear stimuli ($p > .2$ in each test).
