# Supplementary material for: The auditory cortex hosts network nodes influential for emotion processing: An fMRI study on music-evoked fear and joy
Source: PLoS One. 2018 Jan 31;13(1):e0190057. doi: 10.1371/journal.pone.0190057 (PMC5791961; doi:10.1371/journal.pone.0190057)
Supplement: S2 Text — (PDF) [file pone.0190057.s003.pdf]

S2 Text. PPI results for non-auditory seed regions.

We also computed PPI analyses for the remaining (non-auditory) computational hubs indicated by the ECM-analysis (left paracentral lobule and ACC). Although this was not the focus of our study, and although we did not have a-priori hypotheses regarding the connectivity of these structures, results are provided here to generate hypotheses for future studies. The *left paracentral lobule* showed a significantly stronger functional connectivity during the fear (compared with the joy) condition with visual cortex (V1 - V4), right parahippocampal cortex, precuneus, cingulate cortex, and bilateral cerebellum (Supporting Information S2 Table). The pregenual *ACC* showed significantly stronger functional connectivity during the joy condition with the precuneus, superior parietal lobule bilaterally (postcentral gyrus), right inferior parietal lobule, motor, premotor, and supplementary motor cortex, as well as with the cerebellum bilaterally (Supporting Information S2 Table).
