# Supplementary material for: The auditory cortex hosts network nodes influential for emotion processing: An fMRI study on music-evoked fear and joy
Source: PLoS One. 2018 Jan 31;13(1):e0190057. doi: 10.1371/journal.pone.0190057 (PMC5791961; doi:10.1371/journal.pone.0190057)
Supplement: S1 Table — (PDF) [file pone.0190057.s004.pdf]

Composer	Title	ASIN
Joy-evoking		
J.S. Bach	Bourree (Overture No. 1, BWV 1066)	
Anonymous	Entree-Courante	CD-ASIN B0000247QD
N. Paganini	Violin Concerto Nr 1, 3rd movement	
Louis Armstrong	St. Louis Blues	CD.ASIN B000FBG0HG
J. Pastorius	Soul intro ("The Chicken,"	CD-ASIN: B0000C24JN)
New Celtic Dimension	The Lucky Penny	CD-ASIN B000003NHN
F. Canaro	La Punalada	CD-ASIN: B00000DXZQ
anonymus	Irish Jig	
Fear-evoking		
Akihiko Matsumoto et al.	Freezer Burn (from: Resident Evil Outbreak)	B00019257G
Danny Elfman	The Killing	B00000JC9R
Michael Giacchino	Monsters Are Such Interesting People (from: Lost)	B000EHSVDM
Michael Giacchino	Just Another Day on the Beach (from: Lost)	B000I2IQ9M
Michael Giacchino	Charlie's Dream (from: Lost)	B000I2IQ9M
Takeshi Miura et al.	Pulsating Right Arm (from: Biohazard Code)	B00005HWMB
Seiko Kobuchi	Boss Battle (from: Biohazard Zero)	n/a
Masami Ueda & Saori Maeda	Cold Sweat (from: Biohazard 3)	B000058A7Y

S1 Table. List of stimuli.
