# Supplementary material for: The auditory cortex hosts network nodes influential for emotion processing: An fMRI study on music-evoked fear and joy
Source: PLoS One. 2018 Jan 31;13(1):e0190057. doi: 10.1371/journal.pone.0190057 (PMC5791961; doi:10.1371/journal.pone.0190057)
Supplement: S2 Table — (PDF) [file pone.0190057.s005.pdf]

Seed region	Functionally connected structures	MNI coord.	cluster size (mm^3)	z-value: max (mean)
l pregenual cingulate cortex				
	precuneus	0 -54 58	14931	4.79 (3.49)
	l postcentral g.	-51 -16 55	3159	5.23 (3.57)
	r postcentral g.	60 -18 37	1863	4.71 (3.54)
	l p.t.	-54 -33 19	945	4.17 (3.44)
	r fusiform g.	33 -42 -23	2187	4.07 (3.43)
	r calcarine s.	3 -72 10	45495	5.14 (3.58)
	l occipital g.	-42 -81 13	378	4.18 (3.44)
	l cerebellum	-24 -51 -44	675	3.84 (3.41)
Paracentral lobule				
	MCC	-8 3 40	1620	-4.17 (-3.42)
	precuneus	6 -54 73	594	-4.06 (-3.46)
	cuneus	3 -78 37	54081	-5.29 (-3.58)
	r cerebellum	15 -81 -29	567	-3.87 (-3.27)

PPI results with seeds in the anterior cingulate cortex and in the paracentral lobule (corrected for multiple comparisons, $p < .001$). Positive z-values indicate stronger functional connectivity between a seed region and a functionally connected structure during the joy (compared to the fear) condition, negative z-values indicate stronger functional connectivity between a seed region and a functionally connected structure during the fear (compared to the joy) condition. Abbreviations: g.: gyrus; l: left; MCC: middle cingulate cortex; p.t.: planum temporale; r: right; s.: sulcus.

S2 Table. PPI results for non-auditory seed regions.
